# SQSTM1/p62 as a therapeutic target in cancer

**DOI:** 10.1080/27694127.2022.2037050

**Published:** 2022-03-24

**Authors:** Hyeong-Reh C. Kim, Abdo J. Najy, Seongho Kim, Yong Tae Kwon

**Affiliations:** a Department of Pathology; b Department of Oncology; c Biostatistics and Bioinformatics Core,Wayne State University School of Medicine, Karmanos Cancer Institute, Detroit, MI, 48201,USA; d Cellular Degradation Biology Center and Department of Biomedical Sciences, College of Medicine, Seoul National University, Seoul, Korea; e AUTOTAC Bio Inc., Changkkyunggung-ro 254, Jongno-gu, Seoul, 03080, Korea

**Keywords:** apoptosis, autophagy/macroautophagy, CASP8 aggresome, ER stress, small molecule ligand, SQSTM1/p62

## Abstract

Cell survival depends on dynamic interactions among the signaling pathways that control the endoplasmic reticulum (ER) stress response, macroautophagy/autophagy and apoptotic cell death. Our recent study reported an association of cytoplasmic SQSTM1/p62-mediated autophagy with disease progression and therapy resistance in head and neck squamous cell carcinoma (HNSCC). Synthetic small molecule ligands of SQSTM1 activate autophagic flux by binding the SQSTM1 ZZ domain and promoting self-oligomerization. Importantly, we found that the combination of pharmacological activation of SQSTM1 and therapeutic radiation promotes formation of ubiquitinated CASP8 (caspase 8) aggresomes that lead to apoptotic cell death of HNSCC. This finding suggests the potential for the development of a novel therapeutic strategy involving pharmacological activation of SQSTM1 in intrinsically apoptosis-resistant and therapy-resistant cancer cells.

## Potential of a small-molecule ligand of SQSTM1 as a therapeutic tool in intrinsically apoptosis-resistant cancers

**Figure 1.**
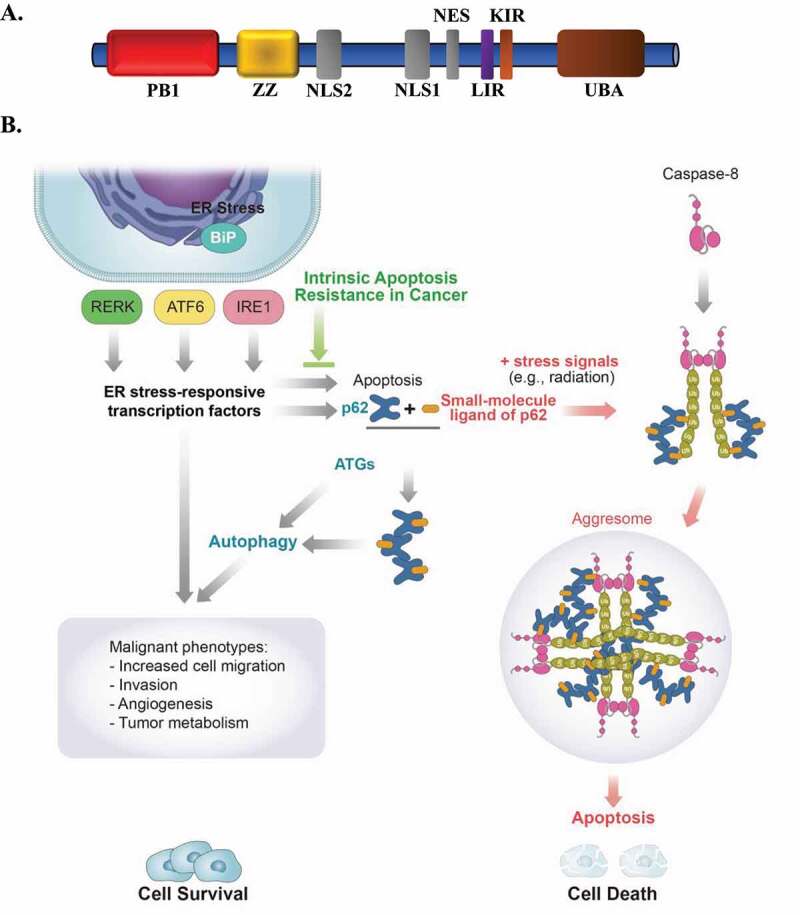
ER stress pathways and small-molecule ligand of SQSTM1-induced apoptosis in combination with ionizing radiation. (**A**) Schematic representation of Phox-and-Bem1 (PB1), ZZ, nuclear localization signal (NLS), nuclear export signal (NES), and LC3-interacting region (LIR), KEAP1-interacting region (KIR), and ubiquitin-associated (UBA) domains of SQSTM1. (**B**) A graphic illustration of ER stress pathways and small-molecule ligand of SQSTM1-mediated CASP8 activation upon irradiation in intrinsically apoptosis-resistant cancer cells.

A critical goal in developing cancer treatments is the identification of druggable targets that selectively exploit cancer cell vulnerabilities. Considering the evidence of increased autophagic flux as a key mechanism for cancer cell survival, autophagy has emerged as an attractive therapeutic target in cancer. Autophagy inhibitors such as chloroquine or hydroxychloroquine have shown only minor benefits in promoting tumor cell death in clinical trials. Their off-target effects and side effects associated with systemic inhibition of autophagy remain a concern. Efforts also have been made to manipulate the balance between autophagy and apoptosis, as their regulation is closely connected. Yet, this approach is faced with the challenge of inducing apoptotic cell death because the upstream signaling cascades in major apoptotic pathways, such as mitochondrial or cell death receptor pathways, are often inhibited in cancer cells. Our recent study [1] proposes small-molecule ligands of SQSTM1 as a therapeutic tool to convert survival autophagy to apoptotic cell death in ER-stressed and apoptosis-resistant cancer cells. We previously reported that the N-terminally arginylated form of the ER chaperone HSPA5/GRP78/BiP binds to the SQSTM1 ZZ domain (Fig. 1A) for its autophagic degradation. Subsequently, we have developed small-molecule ligands to the ZZ domain of SQSTM1 including YOK-1104, used in our recent study, and demonstrated the ability of synthetic small-molecule ligands of SQSTM1 to induce SQSTM1 self-oligomerization, autophagic sequestration of its cargoes, and autophagosome biogenesis. In the presence of additional stress stimuli such as irradiation, which induces ubiquitination of Caspase8 (CASP8), pharmacologically activated SQSTM1 serves as a focal point for its interaction with ubiquitinated CASP8 and facilitates CASP8^+^ SQSTM1^+^ aggresome-like structures leading to apoptotic cell death (Fig. 1B). This process occurs in cancer cells that are resistant to both intrinsic mitochondrial and extrinsic cell death receptor pathways. The SQSTM1 ligands-initiated CASP aggresomes may represent a novel apoptosis signaling complex, bypassing defects in the formation of “apoptosome”-like structures triggered by CYCS/cytochrome c release from the mitochondria or the death-inducing signaling complex (DISC) involving death receptors on the cell surface.

## Is SQSTM1 an attractive therapeutic target in cancers?

**Figure 2.**
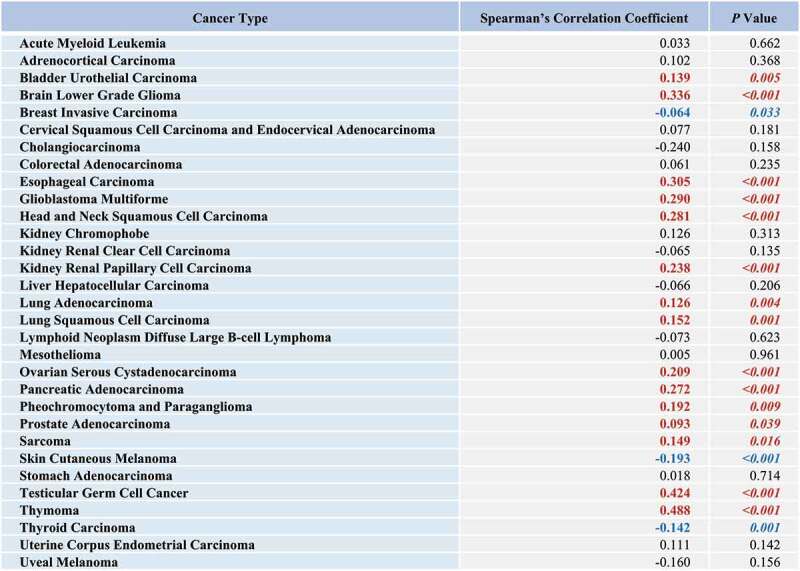
Association between ER stress and SQSTM1 expression. The correlations of RNA expression levels between *SQSTM1* and ER stress-related genes for each cancer type were computed using Spearman’s correlation coefficients. The Cancer Genome Atlas (TCGA) provisional RNA expression data were obtained through cBioPortal. The expression levels of 16 ER stress-related transcription factors (*XBP1, ATF4, ATF6, DDIT3/GADD153/CHOP, NFYA/CBF, SREBF1/SREBP1, YY1, ESRRA, ATF3, JUN, FOXO1, IRF1, TP53/p53, NFKB1, NFE2L2/NRF2/ARE*, and *HNF4A*) were obtained by uniformly summing the expression levels of all 16 genes. The significant correlations (p<0.05) are indicated with italic and bold fonts. Positive and negative significant associations between SQSTM1 and ER stress-induced TFs expression are indicated with red and blue colors, respectively.

Increasing evidence demonstrates robust ER stress signaling in human cancers. Cancer cells are constantly subjected to intense stressors such as chromosomal aberrations, genetic mutations, increased glucose metabolism, free radicals, and imbalance in Ca^2+^ homeostasis. As tumors grow and invade into other tissues, they are exposed to hostile microenvironments including cell-ECM tension, hypoxia, nutrient deprivation, acidosis, and oxidative stress. These stimuli disrupt ER homeostasis and cause protein misfolding. Accumulation of misfolded proteins and ER stress results in the activation of unfolded protein response (UPR) signaling through three major sensors of ER stress: ERN1/IRE1α (endoplasmic reticulum to nucleus signaling 1), EIF2AK3/PERK (eukaryotic translation initiation factor 2 alpha kinase 3) and ATF6/ATF6α (activating transcription factor 6). Activation of these stress sensors, controlled by the ER-resident chaperone molecule HSPA5/GRP78/BiP, results in transcriptional upregulation of its downstream genes via several transcription factors. Whereas the UPR is initially utilized to resolve ER stress and support cell survival, sustained signals trigger apoptotic signaling pathways. Besides apoptosis, the UPR also induces autophagy involving transcriptional upregulation of autophagy-related genes (ATGs) and the autophagic receptor SQSTM1. While cellular homeostasis is maintained by the balance between autophagy and apoptosis, cancer cells often acquire genetic alterations leading to apoptosis resistance and adapt to these potentially fatal stress signals, where ER stress-induced autophagy contributes to cancer cell survival under ER stress conditions. To survey the relevance of SQSTM1 expression levels to ER stress responses in cancers, we analyzed RNA expression levels of *SQSTM1* and 16 transcription factors (TFs), known to be involved in ER stress-regulated gene expression, as a signature of ER stress. TCGA provisional data analysis showed a significant positive association between SQSTM1 and ER stress-induced TFs expression in 15 cancer types (Fig. 2), supporting the notion that SQSTM1 might be an attractive therapeutic target in some ER-stressed cancers, if not all. Perhaps more importantly than the increased expression levels of SQSTM1, SQSTM1 ligand-mediated apoptosis occurs in cells with elevated autophagic flux and thus SQSTM1 ligands might specifically target malignant cells while avoiding toxicity in normal cells.

## Challenges and future study

We postulate that aberrant UPR signaling, often seen in cancer cells, upregulates SQSTM1 expression and autophagic flux among other dysregulation of cellular processes (Fig. 1B). Our novel approach aims to convert the autophagic receptor function of SQSTM1 for cancer cell survival to the formation of CASP aggresomes for cancer cell death in conjunction with irradiation. We surmise that other potent stress signaling leading to ubiquitination of caspases may also cooperate with SQSTM1 ligands and result in the formation of CASP^+^ SQSTM1^+^ aggresome-like structures. A multimodal treatment protocol with the right combination, optimized delivery timing/sequence, and the most effective delivery route for the formation of CASP aggresomes *in vivo* remain to be established. Potential beneficial or harmful effects of sustained pharmacological activation of SQSTM1 in normal cells also remain to be fully investigated *in vivo*.
